# Sodium channel expression in the ventral posterolateral nucleus of the thalamus after peripheral nerve injury

**DOI:** 10.1186/1744-8069-2-27

**Published:** 2006-08-17

**Authors:** Peng Zhao, Stephen G Waxman, Bryan C Hains

**Affiliations:** 1 Department of Neurology and Center for Neuroscience and Regeneration Research, Yale University School of Medicine, New Haven, CT 06510, USA; 2 Rehabilitation Research Center, VA Connecticut Healthcare System, West Haven, CT, 06516, USA

## Abstract

Peripheral nerve injury is known to up-regulate the expression of rapidly-repriming Nav1.3 sodium channel within first-order dorsal root ganglion neurons and second-order dorsal horn nociceptive neurons, but it is not known if pain-processing neurons higher along the neuraxis also undergo changes in sodium channel expression. In this study, we hypothesized that after peripheral nerve injury, third-order neurons in the ventral posterolateral (VPL) nucleus of the thalamus undergo changes in expression of sodium channels. To test this hypothesis, adult male Sprague-Dawley rats underwent chronic constriction injury (CCI) of the sciatic nerve. Ten days after CCI, when allodynia and hyperalgesia were evident, *in situ *hybridization and immunocytochemical analysis revealed up-regulation of Nav1.3 mRNA, but no changes in expression of Nav1.1, Nav1.2, or Nav1.6 in VPL neurons, and unit recordings demonstrated increased background firing, which persisted after spinal cord transection, and evoked hyperresponsiveness to peripheral stimuli. These results demonstrate that injury to the peripheral nervous system induces alterations in sodium channel expression within higher-order VPL neurons, and suggest that misexpression of the Nav1.3 sodium channel increases the excitability of VPL neurons injury, contributing to neuropathic pain.

## Background

Peripheral nerve injury can result in the development of chronic pain that is associated with hyperexcitability of sensory neurons within the dorsal root ganglia (DRG) [1,2] and the spinal cord dorsal horn [3-5]. Changes in sodium channel expression are known to contribute to neuronal hyperexcitability, and to reductions in behavioral nociceptive thresholds after nerve injury. It is now well-established that peripheral axotomy and chronic constriction injury (CCI) trigger upregulated expression of the Nav1.3 sodium channel within DRG neurons [6-8] and that CCI is followed by upregulation of Nav1.3 within nociceptive dorsal horn neurons [9]. This is functionally important because Nav1.3 produces a persistent current [10] and a ramp response which amplifies small depolarizations close to resting potential, and reprimes rapidly from inactivation [11,12], thereby contributing to hyperexcitability of these neurons [9].

Questions remain regarding molecular changes in supraspinal sensory neurons after nerve injury. Of particular interest is the ventral posterolateral (VPL) nucleus of the thalamus which receives input from spinal sensory neurons, and is involved in sensory-discriminative aspects of pain processing [13]. Previous work has demonstrated that VPL neurons sensitize to mechanical and thermal stimuli after peripheral neuropathy [14], and that NMDA blockade can decrease nociceptive transmission [15]. However, whether there are changes in sodium channel expression within the thalamus that might contribute to neuronal hyperresponsiveness after injury is not yet known.

In this study we asked whether peripheral nerve injury can also trigger supraspinal changes in sodium channel expression within the thalamus. We hypothesized that upregulated expression of Nav1.3, and possibly other isoforms, occurs in third-order VPL neurons after peripheral nerve injury.

## Results

### Behavioral testing

Testing of behavioral nociceptive thresholds was performed to confirm that animals had developed pain-related behaviors following CCI, at the time of histological or electrophysiological analysis. Ten days following CCI, animals demonstrated significantly reduced hindlimb mechanical thresholds on the ipsilateral side (4.1 ± 2.5 g) when compared to the contralateral side (18.8 ± 4.7 g) or sham-operated animals (21.9 ± 2.6 g) (data not shown), indicating the development of mechanical allodynia. Thermal paw withdrawal latencies were also significantly reduced for the ipsilateral hindlimb 10 d after CCI (4.3 ± 2.0 sec) relative to the contralateral side (9.8 ± 2.4 sec) or sham-operated animals (10.2 ± 2.6 sec) (not shown), indicating the development of thermal hyperalgesia.

### Extracellular unit recordings

**Figure 1:**
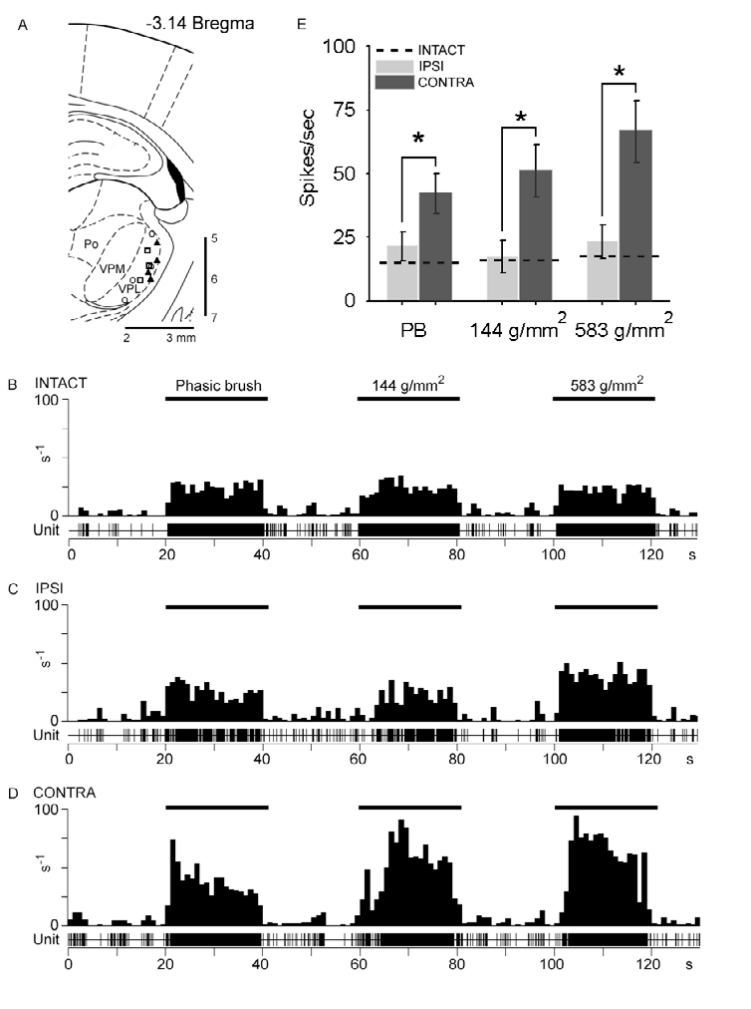
Two-dimensional distribution of 10 histologically identified recording sites plotted on a schematic diagram [16] of the ventrobasal complex of the thalamus corresponding to bregma -3.14 mm, which delineates the posterior nucleus group (Po), ventral posteromedial nucleus (VPM), and VPL. Units from intact control (open circles), 10 days after chronic constriction injury (CCI) ipsilateral side (open squares), and CCI contralateral side (filled triangles) groups are shown (A). All units used in this analysis were confined to the VPL. Representative extracellular multireceptive unit recordings plotted as peristimulus time histograms as well as unit activity are shown for intact (B), and ipsilateral (C) and contralateral (D) sides after CCI in response to phasic brush, 144 g/mm^2 ^press, and 583 g/mm^2 ^pinch, stimulation (10 sec) of peripheral receptive fields located on the corresponding hindpaw. Quantification (E) of spikes/second show that 10 days after CCI, on the contralateral side, evoked discharge rates were significantly (*p < 0.05) elevated in response to all peripheral stimuli compared to intact animals and the ipsilateral side of CCI animals.

Examination of sections corresponding to the ventrobasal complex of the thalamus at bregma -3.14 mm confirmed that the tip of the recording electrode was within the VPL (Figure 1A). Representative unit recording locations are shown for intact as well as CCI animals for ipsilateral and contralateral sides at 10 days after injury, superimposed on a schematic diagram of the thalamus [16]. Typically the track of the electrode passed through the hippocampus and VPM. All units analyzed were located within the atlas boundaries of the VPL.

Representative peristimulus time histograms from an intact animal (Figure 1B), as well as from sides ipsilateral (Figure 1C) and contralateral (Figure 1D) to the CCI 10 days after injury, show that in comparison to MR units recorded from intact and the ipsilateral VPL after CCI, units recorded from the contralateral side exhibited elevated evoked firing rates. Quantification of mean evoked rates (Figure 1E) revealed that in intact animals, mean evoked discharge rates to phasic brush (17.5 ± 2.4 Hz), 144 g/mm^2 ^(19.1 ± 3.6 Hz), and 583 g/mm^2 ^(22.1 ± 4.4 Hz) compressive stimuli were in accordance with previously published reports [17]. Ten days after induction of CCI, on the side ipsilateral to the CCI, evoked responses to brush (23.9 ± 4.2 Hz), 144 g/mm^2 ^(19.4 ± 4.2 Hz), and 583 g/mm^2 ^(24.5 ± 5.9 Hz) were not significantly different from intact animals. In contrast, on the contralateral side, discharge rates during application of brush (42.1 ± 4.8 Hz), 144 g/mm^2 ^(50.6 ± 11.4 Hz), and 583 g/mm^2 ^(69.2 ± 13.6 Hz) were significantly (p < 0.05) increased compared to intact and ipsilateral CCI units.

### Spontaneous thalamic activity independent of spinal afferent barrage

**Figure 2:**
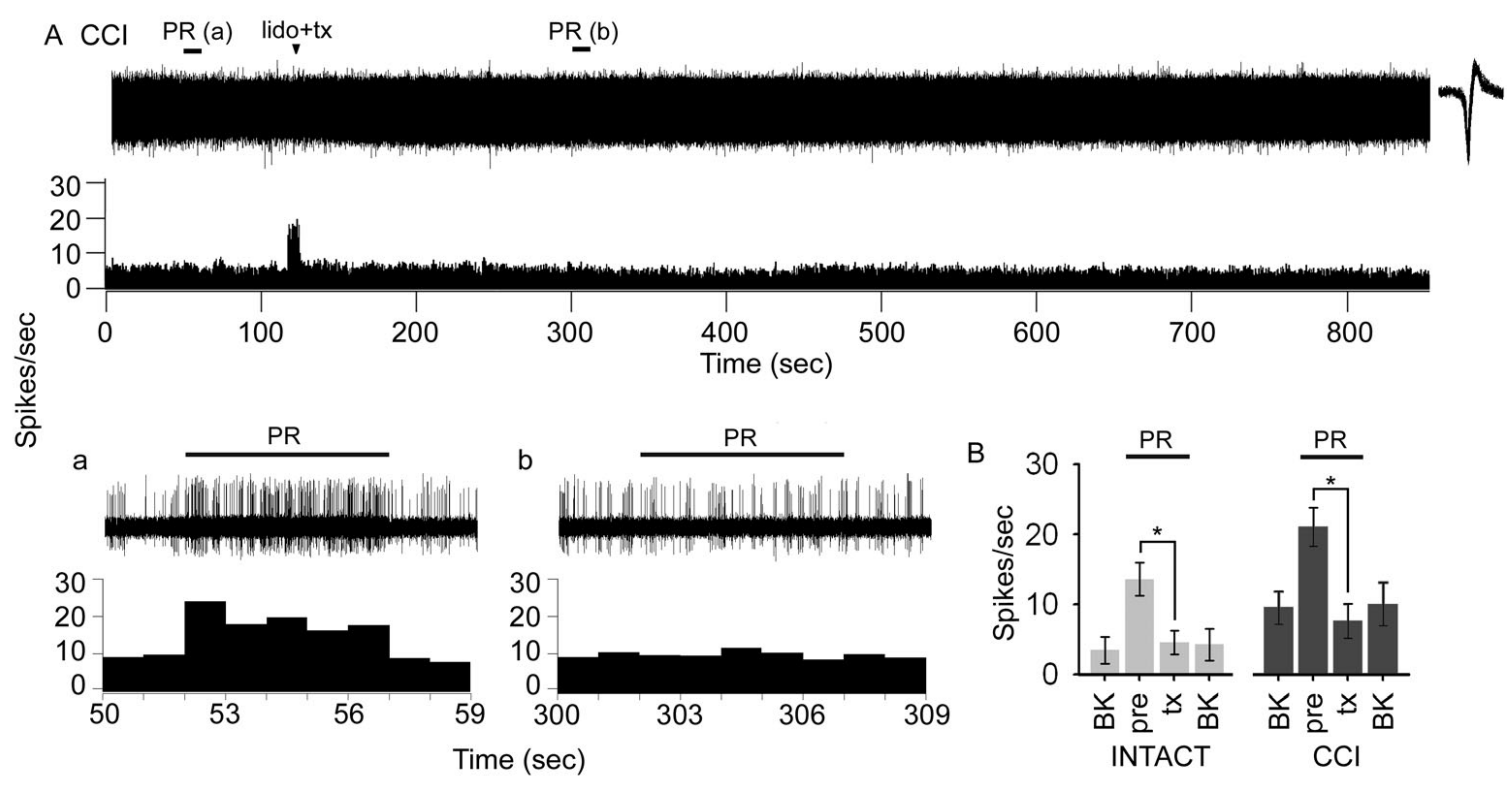
Representative recording of spontaneous and evoked activity of a contralateral VPL neuron with a hindlimb receptive field demonstrated spontaneous discharge 10 days after CCI (A). The VPL unit was continuously recorded, and the spinal cord was acutely transected at T6 following application of 2% lidocaine (lido+tx, at t = 120 s). The corresponding unit waveform is shown. Spontaneous background (BK) activity and evoked responses to brush and press (PR, bar) stimuli are shown on an expanded time scale before (a, t = 50–59 sec) and after (b, t = 300–309 sec) cord transection. In CCI animals, spontaneous firing of VPL neurons was unaffected and occurred at a frequency of 5–12 spikes/s following cord transection, but no evoked responses to PR could be elicited (b). Quantification (B) revealed that evoked responses could no longer be elicited after cord transection in intact and CCI (contralateral) groups, and that background activity remained significantly (*p < 0.05) elevated in CCI animals before (pre) and after interruption (tx) of ascending afferent barrage compared with intact animals.

Recordings from VPL neurons with hindlimb receptive fields demonstrated a high rate of background firing 10 days after CCI. To ascertain whether this high rate of firing was the result of increased afferent barrage from sites below the VPL, we recorded from VPL neurons before and after application of 2% lidocaine and subsequent spinal cord transection at T6. In a representative record from an animal with CCI (Figure 2A), spontaneous background activity was present in the contralateral VPL and occurred at 5–11 Hz (Figure 2B), and a response was evoked upon brush and press stimulation of the hindpaw (Figure 2a). Topical lidocaine application and cord transection (at t = 120 s) at T6 had no effect on spontaneous firing rates of thalamic units which remained high (5–12 Hz) (Figure 2B), although it abolished the evoked response to peripheral stimulation of the hindpaw (Figure 2b). In this series of experiments, after blockade of evoked afferent activity, increased spontaneous activity of VPL units was present for the duration of the experiment (up to 1450 sec). Compared to intact animals, background activity was significantly higher in the contralateral VPL of CCI animals (Figure 2B).

### In situ hybridization

**Figure 3:**
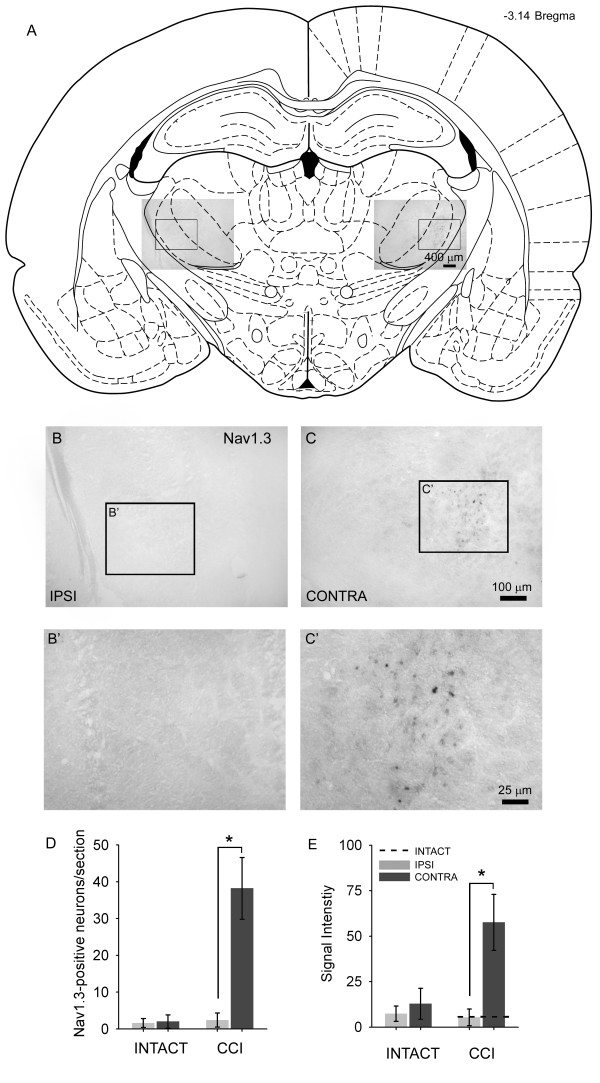
Schematic representation of a coronal brain section corresponding to bregma -3.14 [16] showing the location of the VPL and image locations. Ten days after CCI, representative images from *in situ *hybridization for detection of Nav1.3 mRNA are shown for the ipsilateral (B) and contralateral (C) sides. On the ipsilateral side, only very light Nav1.3 signal was detectable. On the contralateral side, punctuate Nav1.3 signal was present in CCI animals. Higher magnification insets are shown for the ipsilateral (B') and contralateral (C') sides. Quantification of the number of Nav1.3-positive cells exhibiting a neuronal morphology from each group is shown in (D). Compared to intact animals, and to the ipsilateral side after CCI, the contralateral VPL exhibited a significantly (*p < 0.05) increased number of Nav1.3-positive profiles after CCI. Nav1.3 signal intensity was very low in intact animals, and on the ipsilateral side after CCI, whereas on the contralateral side signal intensity was significantly increased after CCI.

*In situ *hybridization studies were performed on intact and CCI brains for detection of mRNA for neuronal sodium channels Nav1.1, Nav1.2, Nav1.3, and Nav1.6 in the VPL. Sections from the ipsilateral and contralateral sides 10 days after CCI probing for Nav1.3 are shown in Figure 3. A coronal brain slice corresponding to bregma -3.14 mm illustrates the location of image fields (Figure 3A) and location of the VPL. On the side ipsilateral to the CCI, very little hybridization signal for Nav1.3 is detectable (Figure 3B). On the contralateral side, however, Nav1.3 hybridization signal was clearly present within small (5–20 μm diameter) neurons in the VPL (Figure 3C). Signal was punctuate and easily discernable from background. Neurons expressing Nav1.3 mRNA were localized to the VPL exclusively, and no signal was observed in the VPM, intralaminar, or adjacent thalamic nuclei. The number of Nav1.3-positive neurons ranged from 22–54 (mean 38.2 ± 8.4 neurons) per section on the contralateral side, significantly higher compared to 0–4 (2.4 ± 1.9 neurons) in intact animals and in CCI animals on the side ipsilateral to CCI (1.9 ± 1.2 neurons) (Figure 3D). Signal intensity within Nav1.3-positive neurons was significantly elevated only on the side contralateral to CCI (Figure 3E). In intact animals, the signal intensity was relatively low (7.4 ± 4.2 arbitrary units), and was not different from the ipsilateral side of CCI animals (5.4 ± 4.6 units). In contrast, the contralateral side of CCI rats displayed significantly increased Nav1.3 signal intensity (57.6 ± 15.4 units).

In intact animals, Nav1.1, Nav1.2, and Nav1.6 were constitutively expressed within the VPL. Nav1.1 was expressed on both ipsilateral (mean 13.9 ± 4.0 neurons per section) and contralateral (16.1 ± 7.1 neurons) VPL after injury, which was not changed relative to intact (15.2 ± 4.4 neurons) animals. Similarly, Nav1.2 was expressed but was not different from control (31.2 ± 3.0 neurons) in either the ipsilateral (34.1 ± 2.6 neurons) or contralateral (30.0 ± 4.7 neurons) sides after CCI. Differences in Nav1.6 expression were not detected relative to intact animals (23.6 ± 4.9 neurons) in either ipsilateral (26.1 ± 5.2 neurons) or contralateral (22.5 ± 6.5 neurons) sides after CCI.

**Figure 4:**
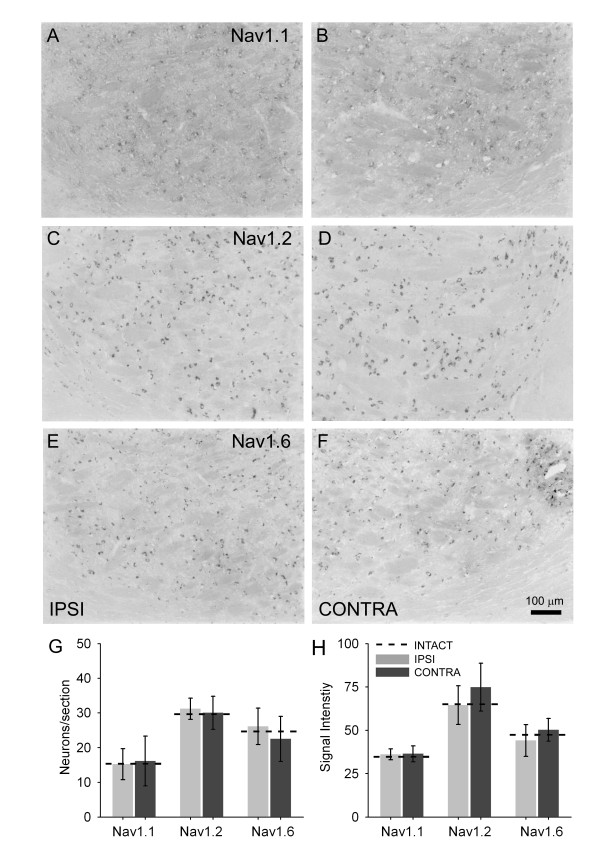
Representative images showing Nav1.1 (A, B), Nav1.2 (C, D), and Nav1.6 (E, F) mRNA transcripts within regions corresponding to the ipsilateral (A, C, E) and contralateral (B, D, F) VPL in coronal brain sections collected at bregma -3.14 10 days following CCI. Signal was detectable in cells exhibiting a neuronal morphology. Quantification of the number of neurons per section expressing each sodium channel isoforms (G) did not reveal any significant differences in ipsilateral or contralateral expression after CCI when compared to intact animals. Quantification of sodium channel *in situ *signal intensity (H) showed moderate expression of all channel isoforms in intact animals, and that 10 days after CCI no isoforms exhibited changes in signal intensity.

In situ hybridization for Nav1.1 revealed widespread punctuate staining. Quantification of signal intensity in ipsilateral (36.1 ± 3.2 arbitrary units) (Figure 4A) and contralateral (36.4 ± 4.6 units) (Figure 4B) revealed no significant difference 10 days following CCI. Similarly, Nav1.2 signal intensity between ipsilateral (64.5 ± 11.1 units) (Figure 4C) and contralateral sides (74.8 ± 13.8 units) (Figure 4D) were not significantly different after CCI. Finally, Nav1.6 signal intensity was not significantly different between ipsilateral (44.1 ± 9.2 units) (Figure 4E) and contralateral (50.2 ± 6.5 units) (Figure 4F) sides after CCI. Compared to intact animals, no significant differences were observed after CCI (Figure 4H).

## Discussion

In the present study we confirm that peripheral nerve injury (CCI) results in long-distance changes in responsiveness to peripheral stimulation in third-order pain-processing neurons of the ventral posterolateral (VPL) nucleus of the thalamus that receive inputs from the injured contralateral sciatic nerve in animals that display lowered behavioral nociceptive thresholds [14,18]. These changes include increased rates of firing in response to natural peripheral stimuli equivalent to light touch, pressing of the skin, and pinching of the skin. Our new data also document the abnormal expression of the Nav1.3 voltage-gated sodium channel transcripts in the VPL during this time of neuronal hyperresponsiveness and reduced nociceptive thresholds. We observed no changes in the expression of neuronal sodium channels Nav1.1, Nav1.2, or Nav1.6 in VPL neurons, although we can not rule out a contribution of other channels that could have an affect on firing thresholds [19,20]. Our results demonstrate for the first time, that changes in sodium channel expression within the thalamus are associated with abnormal sensory processing and chronic neuropathic pain after CCI.

The abnormal expression of Nav1.3 in third-order neurons suggests a mechanism whereby injury to a peripheral nerve can propagate pathological molecular changes to sequentially-ordered upstream targets. Second-order dorsal horn nociceptive neurons receive input from the periphery via the dorsal root ganglia, and project rostrally to third-order neurons within a pain-signaling pathway, located within the ventrobasal complex of the thalamus. Most of these spinothalamic projections terminate in the VPL, and this pathway underlies most of the transmission of nociceptive information from the periphery to the brain [21]. No changes in expression of Nav1.3 were observed in other thalamic nuclei, such as the intralaminar nuclei [22], which is important in processing affective components of pain. This could be related to the relatively few number of projections from the lumbar spinal cord, or low levels of Nav1.3 transcript upregulation after CCI. We have previously demonstrated that Nav1.3 contributes directly to hyperresponsiveness of second-order dorsal horn neurons after CCI [9], and that knock-down of Nav1.3 expression with intrathecally-delivered antisense oligonucleotides that selectively target Nav1.3 mRNA can reduce Nav1.3 expression in NK1R-positive dorsal horn nociceptive neurons, quiet hyperresponsive dorsal horn neurons, and return behavioral nociceptive thresholds to near-normal levels.

In this study, we document Nav1.3 misexpression in third-order neurons after nerve injury. The number of Nav1.3-expressing neurons is quite low, and may reflect the fact that only a subset of neurons receives pathological input from dorsal horn neurons rendered hyperresponsive by DRG efferents. Through FosB immunoreactivity, the number of neurons of the VPL that are responsive to noxious stimulation has been studied and is also low [23]; the number of Nav1.3-positive neurons in our study closely approximates this number.

The factors that drive expression of Nav1.3 in these neurons are not yet clear, and a number of possibilities exist. First, if we assume that Nav1.3 enables a neuron to fire at higher-than-normal frequencies, it is possible that VPL neurons are upregulating Nav1.3 in order to accommodate high-frequency information received from the spinal cord. As soon as 3 days after injury, ectopic and inappropriate discharges originate in the injured axons and their cell bodies within the DRG [1,24-26]. This abnormal firing could drive dorsal horn neurons to relay higher frequency afferent information supraspinally towards the VPL, which in turn also become hyperresponsive in response to increased drive and upregulate Nav1.3 to accommodate higher than normal firing frequencies. Second, VPL neurons may undergo reactive changes that make them hyperresponsive. Abnormal firing has been shown to originate and persist within the dorsal horn after peripheral injury [3-5,27], as well as the thalamus after spinal cord injury after interruption of spinal afferent barrage [17]. Activity-dependent central sensitization can outlast the conditioning stimulus for hours [28]. Third, while the up-regulated expression of Nav1.3 within DRG neurons following axotomy appears to be due in part to deprivation from peripheral pools of neurotrophic factors [29-31], the signals that trigger Nav1.3 upregulation within third-order neurons have not been identified. It is known that after peripheral nerve injury 2-deoxyglucose metabolic activity [32] and regional blood flow [33] are increased in the thalamus, and that changes in cannabinoid receptors [34] and monoamine release [35] occur, but whether these are linked to Nav1.3 expression is unclear.

Nav1.3 recovers from inactivation rapidly and produces a depolarizing response to small stimuli close to resting potential, and produces a persistent current – increasing the excitability of cells that express Nav1.3 [7,10-12]. The Nav1.3 sodium channel has been linked to pain-related phenomena in a variety of model systems. Increased expression of Nav1.3 occurs in DRG and dorsal horn neurons following injury to the sciatic nerve [6,7,9,36,37], and while expression of Nav1.3 is not increased in axotomized cortical pyramidal neurons [38]. Up-regulation of Nav1.3 expression in DRG neurons is associated with allodynia and hyperalgesia [8]. Similarly, pain after spinal cord injury is ameliorated after knock-down of Nav1.3 [9,17,39].

Sodium channel blockade after peripheral [40] or central [41] injury with systemic lidocaine administration has been shown to be effective in the amelioration of chronic pain. It is not yet known how lidocaine, for example, might affect sodium channel dysregulation after experimental injury, but this is an important question to ask.

## Conclusion

In sum, our findings demonstrate changes in excitability and expression of Nav1.3, but not other neuronal sodium channels (Nav1.1, Nav1.2, Nav1.6), within contralateral VPL neurons following CCI. Together with our earlier results on dorsal root ganglion [8] and dorsal horn neurons [9], these results provide evidence suggesting that dysregulated Nav1.3 expression at both spinal and supraspinal levels after peripheral nerve injury contributes to altered processing of somatosensory information and chronic neuropathic pain.

## Methods

### Animal care

Experiments were carried out in accordance with National Institutes of Health guidelines for the care and use of laboratory animals, and adhered to the guidelines of the Committee for Research and Ethical Issues of International Association for the Study of Pain; all animal protocols were approved by the Yale University Institutional Animal Use Committee. Adult male Sprague-Dawley rats (200–225 g) were used for this study. Animals were housed under a 12 h light-dark cycle in a pathogen-free area with free access to water and food.

### Chronic constriction injury

Rats (n = 24) were deeply anesthetized with ketamine/xylazine (80/5 mg/kg i.p.) and the left sciatic nerve exposed at the mid-thigh level by blunt dissection of the biceps femoris. For chronic constriction injury (CCI) (n = 12), four chromic gut (4-0) ligatures were tied loosely around the nerve, about 1 mm apart, proximal to its trifurcation, as described by [42]. For sham surgery (n = 12), the sciatic nerve was isolated but not ligated. After sham or CCI surgery, the overlying muscles and skin were closed in layers with 4-0 silk sutures and staples, respectively, and the animal recovered on a 30°C heating pad. Postoperative treatments included saline (2.0 cc s.c.) for rehydration, and Baytril (0.3 cc, 22.7 mg/ml s.c.) to prevent urinary tract infection.

### Behavioral analysis

Testing of nociceptive thresholds was performed (n = 6 animals/group) 10 days after sham or CCI surgery to confirm that CCI animals had developed behavioral signs of chronic pain (for all experiments we used only animals that demonstrated the development of chronic pain). After acclimation to the testing area (30 min), mechanical sensory thresholds were determined by paw withdrawal to application of a series of von Frey filaments (Stoelting, Wood Dale, IL, USA) to the glabrous surface of the paw. Following application of calibrated von Frey filaments (0.4–26 g) with enough force to cause buckling of the filament, a modification of the 'up-down' method of [43] was used to determine the value at which paw withdrawal occurred 50% of the time [44], interpreted to be the mechanical nociceptive threshold.

After acclimation to the test chamber, thermal hyperalgesia was assessed by measuring the latency of paw withdrawal in response to a radiant heat source [45]. Animals were placed in Plexiglas boxes on an elevated glass plate under which a radiant heat source (4.7 A) was applied to the glabrous surface of the paw through the glass plate. The heat source was turned off automatically by a photocell upon limb-lift, allowing the measurement of paw withdrawal latency. If no response was detected, the heat source was automatically shut off at 20 s. Three minutes were allowed between each trial and four trials were averaged for each limb.

### Electrophysiology

Animals that had been sham-operated (n = 6), and CCI animals (n = 6) that exhibited reduced behavioral nociceptive thresholds 10 days after injury, underwent extracellular single unit recording according to established methods [9]. The activity of 3–7 units/animal was recorded, yielding 18–42 cells/group. Rats were initially anaesthetized with halothane (4% in induction chamber), and maintained by tracheal intubation (1.1%, 2–2.5 ml tidal volume, 60–70 strokes/min). Halothane anesthesia lasted ~2 h, until the end of each experiment. Rectal temperature was maintained at 37°C.

For unit recording, the head was fixed in a stereotaxic apparatus (Kopf Instruments, Tujunga, CA, USA) and skin incision and a limited craniotomy exposed the brain surface vertical to the recording sites within the thalamus. Neuronal units were isolated bilaterally from the VPL nucleus of the thalamus [stereotaxic coordinates in mm: bregma (-3.30); lateral (2.6); vertical (4.8)]. Extracellular single-unit recordings were made with low-impedance 5 MΩ tungsten insulated microelectrodes (A-M Systems, Carlsborg, WA, USA). Electrical signals were amplified and filtered at 300–3000 Hz (DAM80, World Precision Instruments, Sarasota, FL, USA), processed by a data collection system (CED 1401+; Cambridge Instruments, Cambridge, UK), and stored on a computer (Pentium 4 PC, Dell, Austin, TX, USA) to construct peristimulus time histograms or Wavemark records. The stored digital record of individual unit activity was retrieved and analyzed off-line with Spike2 software (v3.13, Cambridge Electronic Design, Cambridge, UK).

Once a cell was identified by a gentle probing of the body surface, its receptive field was mapped and stimulated by an experimenter blinded to the treatment of the animal. Background (BK) activity was measured followed by cutaneous receptive field mapping with von Frey filaments and/or brief pinches. Receptive fields for VPM units were exclusively mapped to the head, whereas those for VPL units were mapped to the rest of the body. Three mechanical stimuli were routinely applied: (i) phasic brush (PB) stimulation of the skin with a cotton brush; (ii) increasing intensity von Frey filaments (0.39 g; 1.01 g; 20.8 g forces); (iii) pressure (PR), by attaching a large arterial clip with a weak grip to a fold of the skin (144 g/mm^2^); and (iv) pinch (PI), by applying a small arterial clip with a strong grip to a fold of skin (583 g/mm^2^). Multireceptive units were identified by their responsiveness to brush, press and pinch, and with increasing responsiveness to incrementing strength von Frey stimuli. Low threshold or high threshold units were classified as such based on their high (>30%) rate of response to brush, von Frey stimulation or pinch. BK activity was recorded for 20 s and stimuli applied serially for 20 s, separated by 20 s of baseline activity. Care was taken to ensure that the responses were maximal, that each stimulus was applied to the unit's primary receptive field, and that isolated units remained intact and held for the duration of each experiment using Spike2 template matching routines. Neurons responding mainly to joint movement or to probing subcutaneous tissue were excluded from analysis. Evoked responses were calculated by subtracting the prestimulus baseline activity to yield net number of spikes per response.

At the conclusion of recording, a direct current (1 μA for 20 s) was passed through the recording electrode to identify the location of unit recording sites. Recording sites were plotted from 3–4 animals in each group. The brain was removed and fixed in 4% cold buffered paraformaldehyde in PBS for 48 h at 4°C in 30% sucrose before frozen sectioning at 20 μm. Sections were mounted on gelatin/potassium chromium sulfate-coated slides and stained with cresyl violet (0.1%) for visualization and photomicroscopy.

### Blockade of afferent barrage to the thalamus

In a second group, sham-operated animals (n = 4) and animals 10 days after CCI (n = 4) were prepared for standard electrophysiological recording within the VPL. In these experiments, the spinal cord was exposed by laminectomy at the T6 level, and topical application of 2% lidocaine (20 mg/ml, pH 6.5; Abbott Labs, North Chicago, IL, USA) to the dorsal and lateral surfaces of the spinal cord was followed by complete cord transection at the same site with iridectomy scissors at t = 120 sec. Following cord transection, responses to press stimulation of the hindlimb were abolished in all animals. For each animal one unit was isolated in the VPL that had an identifiable contralateral (injury side) hindlimb receptive field. The unit's activity was continuously recorded for the duration of each experiment. Background and evoked activity were recorded before and after lidocaine and transection.

### In situ hybridization

Following perfusion with 4% paraformaldehyde PBS and cryoprotection in 30% sucrose, coronal sections were collected from the brain at levels corresponding to the ventrobasal complex of the thalamus (bregma -3.14 mm) from animals that had been sham-operated (n = 6), and that exhibited reduced behavioral nociceptive thresholds 10 days after CCI (n = 6). Twelve micron transverse cryosections (n = 5 sections/animal) from each group were processed for detection of mRNA for the neuronal sodium channels Nav1.1, Nav1.2, Nav1.3, and Nav1.6 as previously described [46,47], with incubation in 4% paraformaldehyde increased to 12 min and permeabilization with proteinase K reduced to 6 min. DIG-labeled antisense and sense riboprobes were synthesized as previously described by our group [46,47]. Sense riboprobes yielded no signal on *in situ *hybridization (data not shown). Nav1.4 (which is normally expressed within skeletal muscle), Nav1.5 (cardiac muscle), Nav1.7, Nav1.8, and Nav1.9 (normally expressed in peripheral ganglia and not in brain) were not studied.

### Quantitative image analysis

Images were captured with a Nikon Eclipse E800 light microscope equipped with epifluorescence and Nomarski optics, using a Photometrics CoolSnap HQ camera (Roper Scientific, Tucson, AZ) and MetaVue v6.2r6 software (Universal Imaging Corporation, Downingtown, PA). Quantitative analysis was performed by a blinded observer using MetaVue and IPLab Spectrum v3.0 software (Scanalytics, Fairfax, VA) where the number of positively labeled neurons was counted for ipsilateral and contralateral VPL regions. Cells were sampled only if the nucleus was visible within the plane of section and if cell profiles exhibited distinctly delineated borders. Signal intensity of reaction products was determined by software functions. Background levels of signal were subtracted, and control and experimental conditions evaluated in identical manners.

### Statistical analysis

All statistical tests were performed at the alpha level of significance of 0.05 by two-tailed analyses using parametric tests. Pair-wise comparisons were applied with either the paired Student's t-test or the two sample Student's t-test. Data involving multiple timepoints for individual animals was tested for significance using repeated-measure ANOVA. Data management and statistical analyses were performed using SAS (1992) statistical procedures with Jandel SigmaStat (v1.0), and graphed using Jandel SigmaPlot (v7.0) as mean ± standard deviation (S.D.).

## Abbreviations

BK background

CCI chronic constriction injury

DRG dorsal root ganglion

PB phasic brush

PI pinch

PR pressure

VPL ventral posterolateral

VPM ventral posteromedial

## Competing interests

The author(s) declare that they have no competing interests.

## Authors' contributions

PZ assisted with behavioral testing, in situ hybridization, quantification, and in drafting the manuscript. SGN helped conceive the project, guide the studies, and aided in drafting the manuscript. BCH helped conceive the project, develop the experimental methods, perform electrophysiological recordings, analyze the data, and draft the manuscript.
